# Phytic Acid Enhances Biocontrol Activity of *Rhodotorula mucilaginosa* against *Penicillium expansum* Contamination and Patulin Production in Apples

**DOI:** 10.3389/fmicb.2015.01296

**Published:** 2015-11-23

**Authors:** Qiya Yang, Hongyin Zhang, Xiaoyun Zhang, Xiangfeng Zheng, Jingya Qian

**Affiliations:** School of Food and Biological Engineering, Jiangsu UniversityZhenjiang, China

**Keywords:** phytic acid (PA), *Rhodotorula mucilaginosa*, biocontrol, *Penicillium expansum*, apples, patulin

## Abstract

The effect of *Rhodotorula mucilaginosa* in combination with phytic acid (PA) on blue mold decay and patulin contamination of apples was investigated. Results from this study show that different concentrations of PA were effective in reducing the disease incidence of apples and that PA at concentration of 4 μmol/mL, decreased the incidence of blue mold decay in apples from 86.1 to 62.5%, and showed higher control efficacy compared to untreated, control fruit during storage at 20°C. However, *R. mucilaginosa* combined with PA (4 μmol/mL) showed better control efficacy of blue mold decay than *R. mucilaginosa* used as single treatment, the disease incidence was reduced to 62.5% and lesion diameter on apples was reduced to 16.59 cm. In *in vitro* experiments, the addition of PA enhanced the biocontrol effect of *R. mucilaginosa* against the growth of *Penicillium expansum* and reduced patulin level when compared with either *R. mucilaginosa* or PA used separately. *R. mucilaginosa* together with PA, improved the inhibition of patulin production in wounded apples, decreasing the content of patulin by 89.6% compared to the control, under experimental conditions. Both *R. mucilaginosa* and *R. mucilaginosa* in combination with PA degraded patulin *in vitro*. In conclusion, the appropriate combination of *R. mucilaginosa* and PA may provide an effective biocontrol method for reducing postharvest decay of apples.

## Introduction

Patulin (PAT) is a mycotoxin produced by *Penicillium (P.), Aspergillus (A.)*, and *Byssochlamys* species. Among these fungi, *Penicillium expansum* is the most important producer of the mycotoxin that can be detected in fruits and fruit juices (Sommer et al., 1974; Paster et al., 1995). *P. expansum*, which often develops on the surface of healthy fruit, is the predominant postharvest fungus in apples. *P. expansum* produces PAT that is normally associated with fruits infected by microorganisms in postharvest conditions (Riteni, 2003).

The control of diseases in postharvest fruit is based on the use of chemical fungicides, but the progressive loss of their effectiveness and the emergence of resistant pathogens are major concerns, due to the increasing levels of agro-toxic residues (Sugar and Spotts, 1999; Castoria et al., 2001). The need for exploration of alternatives to synthetic fungicides has attracted attention because intensive use of synthetic chemical fungicides may give rise to a number of toxicological problems regarding human safety and effects on the environment (Droby, 2006). Biological control using antagonistic microorganisms is a promising alternative that provides a safe application method for both human health and the ecosystem (Janisiewicz et al., 2000; Usall et al., 2001). The phylloplane yeast *Rhodotorula mucilaginosa* has been reported to control *Botrytis cinerea* on geranium seedlings in combination with fungicides (Buck, 2004). We previously found that *R. mucilaginosa* has biocontrol efficacy against blue mold in apples caused by *P. expansum* (Li et al., 2011). Recently we discovered the efficacy of *Pichia caribbica* in controlling postharvest blue mold in apples and degrading the patulin produced by *P. expansum* (Cao et al., 2013).

Alternatives to chemical control, particularly biological control, are often less effective than many of the commercial fungicides currently in use. Therefore, the efficacy of antagonistic yeasts in controlling postharvest diseases need to be enhanced (Janisiewicz and Korsten, 2002). Substances (organic and inorganic additives) have been applied in combination with biocontrol agents for a synergistic effect (Janisiewicz, 1994; El-Ghaouth et al., 2000).

Phytic acid is a simple ringed carbohydrate with six phosphate groups attached to each carbon. In the plant kingdom PA is the principal storage form of phosphorus, particularly abundant in cereals and legumes (Shamsuddin, 2002). It may provide some health benefits to the human body, however, it is commonly defined as an antinutrient to humans and non-ruminant animals that reduces the availability of nutrients and decreases the absorption of micronutrients (Brankovic et al., 2015; Burgos-Lujan and Tong, 2015). PA is a naturally occurring compound that is non-toxin, biocompatible, and green to the environment (Gupta et al., 2013). Moreover, Du et al. (2012) found that apple juice treated with phytic acid had significantly lower browning formation during processing and subsequent 6 months of storage at room temperature, compared with the control. Similarly, we have found that the combination of *R. mucilaginosa* and PA at the concentration of 4 μmol/mL was the most effective treatment in controlling the natural spoilage of strawberries after storage at 4°C for 20 days followed by 20°C for 5 days (Zhang et al., 2013). However, to our knowledge, there is no information concerning the effects of a combination of antagonistic yeast and PA on the control of the postharvest blue mold decay and patulin production in apples.

The objectives of this study were to evaluate the effects of PA, *R. mucilaginosa* treatment alone or in combination with PA in controlling postharvest blue mold decay, mycelial growth of *P. expansum*, patulin production by *P. expansum* in NYDB media, the degradation of patulin, and in the production of patulin by *P. expansum* in apples.

## Materials and Methods

### Pathogen Inocula

*Penicillium expansum* (preserved in the China General Microbiological Culture Collection Center, No.3.3703) was isolated from infected apples. The culture was maintained on potato dextrose agar media (PDA: extract of boiled potatoes, 200 g; glucose, 20 g; agar, 20 g and distilled water, 1000 mL) at 4°C. Fresh cultures were grown on PDA plates at 28°C before use. Spore suspensions were prepared by removing the spores from the sporulating edges of a 7-day-old culture with a bacteriological loop, and then suspending them in sterile distilled water. Suspensions were filtered through four layers of cheesecloth to remove fungal mycelia and spore concentrations were determined with a hemocytometer, with the concentration adjusted as required by adding sterile distilled water.

### Antagonist Isolation

The yeast antagonist *R. mucilaginosa* (preserved in the China General Microbiological Culture Collection Center, No.3617) was isolated from the surfaces of peach blossoms picked in unsprayed orchards. Subsequently, sequence analysis of the 5.8S internal transcribed spacer (ITS) ribosomal DNA (rDNA) region was used to identify the yeast. *R. mucilaginosa* has been shown to be safe in animal testing, including physiology experiments, acute toxicity studies, and the Ames test (our unpublished data). *R. mucilaginosa* isolates were maintained at 4°C on nutrient yeast dextrose agar (NYDA) medium containing 8 g nutrient broth, 5 g yeast extract, 10 g glucose, and 20 g agar (Sangon Co., Shanghai, China), in 1 L of distilled water. Liquid cultures of the yeast were grown in 250 mL Erlenmeyer flasks containing 50 mL of NYD broth (NYDB) that had been inoculated with a loop of the culture. Flasks were incubated on a rotary shaker at 28°C for 20 h. Following incubation, the cells were centrifuged (TGL-16M Centrifuge, Xiangyi Co., Changsha, China) at 7000 *g* for 10 min and washed twice with sterile distilled water in order to remove the growth medium. Yeast cell pellets were re-suspended in sterile distilled water and adjusted to an initial concentration of 2–5 × 10^9^ cells/mL before being adjusted to the concentrations required for the different experiments.

### Fruit Samples

Apples (*Malus domestica* Borkh, cv. Fuji) were harvested at commercial maturity from an orchard in Yantai, Shandong province, and selected for uniformity of size, ripeness, and the absence of apparent injury or infection. Fruit was selected randomly and disinfected with 0.1% sodium hypochlorite for 1 min, washed with tap water and allowed to air dry at room temperature (20°C).

### Effects of PA at Various Concentrations of Blue Mold Decay of Apples

The apples were wounded (3 mm diameter and approximately 3 mm deep) using a sterile borer. Each wound was treated with 30 μL of PA (Sangon Co., Shanghai, China) at 2, 4, 6, 8, or 10 μmol/mL. Sterile distilled water was used as a control. Two hours later, 30 μL of *P. expansum* suspension (5 × 10^4^ spores/mL) were inoculated into each wound. After air drying, the apples were stored in enclosed plastic trays to maintain a high relative humidity (about 95%), and incubated at 20°C. The percentage of infected fruit was recorded 10 days after inoculation. There were three replicates of 12 pieces of fruit, and the experiment was conducted twice.

### Effects of *R. mucilaginosa* in Combination with PA on Blue Mold Decay of Apples

The surface of apples was wounded with a sterile cork borer (approximately 3-mm-diameter and 3-mm-deep). Each wound was treated with 30 μL of different treatment solution as follows: (1) the cell suspensions of *R. mucilaginosa* alone (1 × 10^8^ cells/mL), (2) the cell suspensions of *R. mucilaginosa* (1 × 10^8^ cells/mL) supplemented with PA at the concentration of 2, 4, 6, 8, and 10 μmol/mL, (3) sterile distilled water as a control. Two hours later, 30 μL of *P. expansum* suspension (5 × 10^4^ spores/mL) was inoculated into each wound. After air drying, the samples were stored in enclosed plastic trays to maintain a high relative humidity (about 95%) and incubated at 20°C in an incubator (Radford Technology Co., Ltd., Ningbo, China). The percentage of infected fruit and lesion diameter of the treated fruit were recorded 10 days after inoculation. There were three replicates of 12 pieces of fruit, and the experiment was conducted twice.

### Effects of *R. mucilaginosa* in Combination with PA on the Mycelial Growth of *P. expansum*

The effects of PA on the mycelial growth of *P. expansum* was assayed in PDA media. 5-mm-diameter and 5-mm-deep disks were cut from potato-dextrose agar (PDA) plates and then 100 μL of 1 × 10^8^ cells/mL of washed cell suspension of *R. mucilaginosa*, or *R. mucilaginosa* supplemented with PA at 4 μmol/mL, or a solution of PA at 4 μmol/mL, or sterile distilled water as a control, was added into each wound site on the PDA plates. After 2 h, 100 μL of a 5 × 10^4^ spores/mL suspension of *P. expansum* was added into each wound. The plates were incubated at 28°C for seven d, after which the colony diameters of *P. expansum* were recorded. There were three replicates per treatment, and the experiments were repeated three times.

### HPLC Analysis of Patulin

Patulin was analyzed as described by Cao et al. (2013). An Agilent Technologies 1100 series system equipped with a quaternary pump and variable wavelength detector (Switzerland) was used. The analytical column was from Zorbax, SB-C_18_ 250 mm × 4.6 mm, 5 μm (US). The mobile phase consisted of acetonitrile/water (10:90 v/v) with flow rate of 1.0 mL/min. The UV detection was performed at 276 nm. The linear fit was: y = 46.379x + 2.0732, Pearson’s coefficient (*R*^2^) = 0.9997. The limit of quantification (LOQ) was 9.6 ng (0.48 μg/ mL), and the limit of detection (LOD) was 3.2 ng (0.16 μg/ mL), with signal/noise (S/N) ratios of 10.2 and 3, respectively. The recoveries were in the range of 95.2–102.6%.

### Effects of *R. mucilaginosa* in Combination with PA on Patulin Production by *P. expansum* in NYDB Media

One milliliter of one of the following solutions was added into different Erlenmeyer flasks (250 mL), each containing 50 mL of NYDB: (1) the cell suspensions of *R. mucilaginosa* alone (1 × 10^8^ cells/mL); (2) PA solution (4 μmol/mL); (3) the cell suspensions of *R. mucilaginosa* (1 × 10^8^ cells/mL) supplemented with PA at 4 μmol/mL and (4) sterile distilled water as a control. The flasks were then inoculated with 1 mL of *P. expansum* (5 × 10^6^ spores), and incubated at 28°C for 72 h with agitation (180 rpm). Following incubation, cells were centrifuged at 7000 *g* for 10 min, and the supernatant was considered to be an enriched sample.

Approximately 5.0 g of sample was added to 10 mL of water, and was extracted with 3 × 25 mL of ethyl acetate by shaking vigorously for 1 min each time. The organic phases were combined and cleaned with 10 mL of 14 g/L sodium carbonate solution by shaking for 10 s. The phases were allowed to separate and the aqueous phase was immediately extracted with 10 mL of ethyl acetate by shaking for 1 min. The combined organic phases were added to five drops of glacial acetic acid. The combined solutions were then evaporated to just dryness in a water bath at 40°C. The residue was immediately dissolved in 1 mL of buffer solution, filter sterilized (Millipore, 0.45 μm) and 100 μL of this solution was injected into the HPLC system to determine the patulin content (Wu et al., 2008).

### Effects of *R. mucilaginosa* in Combination with PA in the Patulin Content Produced by *P. expansum* in Apples

The surface of apples was wounded with a sterile cork borer (approximately 3-mm-diameter and 3-mm-deep). Each wound was treated with 30 μL of either (1) the cell suspensions of *R. mucilaginosa* alone (1 × 10^8^ cells/mL), (2) PA solution (4 μmol/mL), (3) the cell suspensions of *R. mucilaginosa* (1 × 10^8^ cells/mL) supplemented with PA at 4 μmol/mL or (4) sterile distilled water as a control. Two hours later, 30 μL of the *P. expansum* suspensions (5 × 10^4^ cells/mL) was inoculated into each wound. After air drying, the samples were stored in enclosed plastic trays to maintain a high relative humidity (about 95%) and incubated at 20°C for 10 days. After extracting the wounded tissue and a 1 cm margin surrounding the wound using a sterile borer, followed by high-speed homogenization, the apple juice was considered an enriched sample. The sample was processed as described above to determine the patulin content. There were three replicates of 12 pieces of fruit, and the experiment was repeated twice.

### Degradation of Patulin by *R. mucilaginosa*, Used Alone or in Combination with PA *In Vitro*

One milliliter of one of the following solutions was added into different Erlenmeyer flasks (250 mL), each containing 50 mL of NYDB: (1) the cell suspensions of *R. mucilaginosa* alone (1 × 10^8^ cells/mL); (2) the cell suspensions of *R. mucilaginosa* (1 × 10^8^ cells/mL) supplemented with PA at 4 μmol/mL. To each flask was added 1 mg patulin. The flasks were then incubated at 28°C for 24 h with agitation (190 rpm). Following incubation, cells were centrifuged at 7000 *g* for 10 min and the supernatant was considered to be an enriched sample. The patulin content of the samples was determined as described above to test the effects of *R. mucilaginosa* in combination with PA.

### Effects of PA at Various Concentrations on Antagonistic Yeast

The effects of PA on antagonistic yeast was assayed in NYDA media. 100 μL of 1 × 10^4^ cells/mL of washed cell suspension of *R. mucilaginosa* supplemented with PA at the concentration of 2, 4, 6, 8, and 10 μmol/mL, or cell suspension of *R. mucilaginosa* as a control, was added to the NYDA plates. The plates were incubated at 28°C for 2 days, after which the colonies of *R. mucilaginosa* were recorded. There were three replicates per treatment, and the experiments were repeated twice.

### Statistical Analyses

The data were analyzed by analysis of variance (ANOVA) using the statistical program SPSS/PC version II.x, (SPSS Inc. Chicago, IL, USA), and the Duncan’s multiple range test was used to separate the means. In addition, when two group of data were compared, the independent samples *t*-test was applied for means separation. The statistical significance was assessed at a level of 0.05.

## Results

### Effects of PA at Various Concentrations on Blue Mold Decay of Apples

**FIGURE 1:**
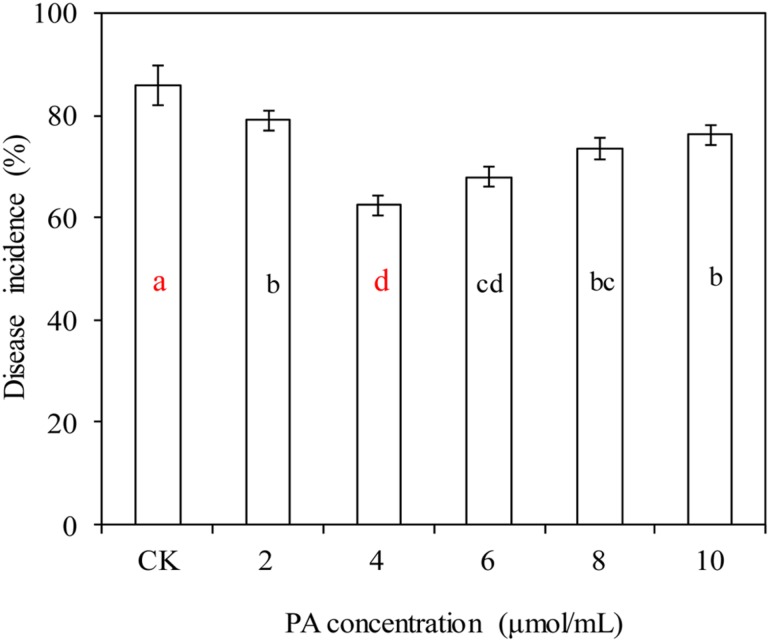
**Effects of phytic acid (PA) at various concentrations on blue mold decay of apples.** Fruit treatments are as follows: CK = sterile distilled water, (2, 4, 6, 8, 10) = PA at different concentrations (2, 4, 6, 8, and 10 μmol/mL). Each value is the mean of two experiments. Bars represent standard deviations. Different letters indicate significant differences (*P* < 0.05) according to the Duncan’s multiple range tests.

Phytic acid, at various concentrations, significantly decreased the incidence of blue mold decay in apples caused by *P. expansum* (*P* < 0.05; **Figure 1**). PA at 4 μmol/mL had the best efficacy, and decreased the incidence of blue mold decay in apples from 86.1% (the control) to 62.5%.

### Effects of *R. mucilaginosa* in Combination with PA on Blue Mold Decay of Apples

**FIGURE 2:**
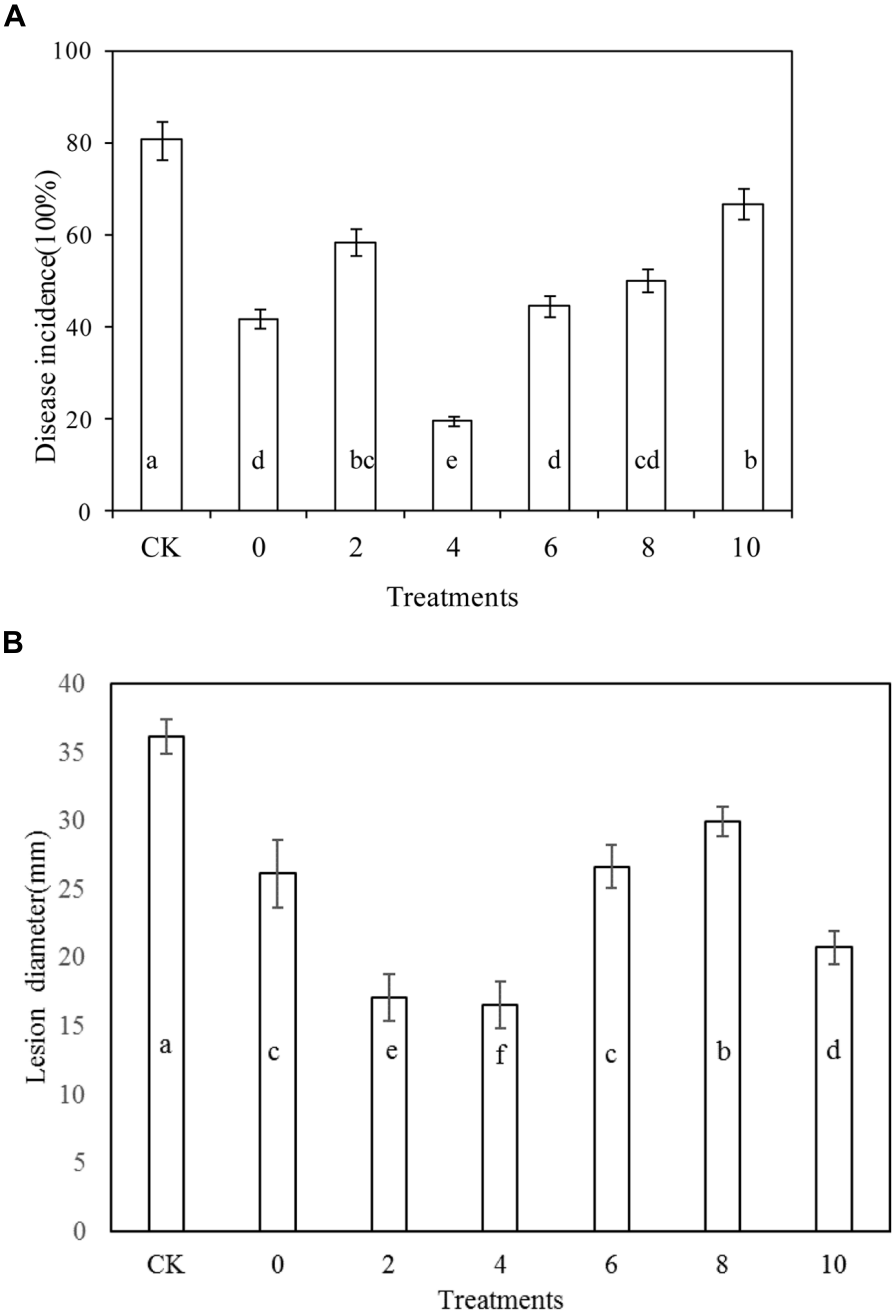
**Effects of *Rhodotorula mucilaginosa* and PA on blue mold decay incidence on apples.** **(A)** Disease incidence, **(B)** Lesion diameter. Fruit treatments are as follows: CK = sterile distilled water, 0 = *R. mucilaginosa* (1 × 10^8^ CFU ml^-1^), (2, 4, 6, 8, 10) = *R. mucilaginosa* (1 × 10^8^ CFU ml^-1^) + PA at different concentrations (2, 4, 6, 8, and 10 μmol/ml). Each value is the mean of two experiments. Bars represent standard deviations. Different letters indicate significant differences (*P* < 0.05) according to the Duncan’s multiple range test and the data from each time point are separated.

*Rhodotorula mucilaginosa* as stand-alone treatment significantly reduced the disease incidence of blue mold decay of apples compared with the control after 10 days storage at 20°C (*P* < 0.05; **Figure 2A**). Similarly, *R. mucilaginosa* in combination with PA at all concentrations tested, (2, 4, 6, 8, and 10 μmol/mL) reduced the disease incidence of blue mold decay of apples to 58.3, 19.4, 44.4, 50, and 66.7%, respectively. The lesion diameter of blue mold decay of the fruit treated with *R. mucilaginosa* in combination with PA at concentrations of 2, 4, and 10 μmol/mL was 17.1, 16.6, and 20.8 cm, respectively, significantly reduced compared with the stand-alone *R. mucilaginosa* treatment (26.2 cm; **Figure 2B**). However, the combined treatments of *R. mucilaginosa* and PA at the concentration of 4 μmol/mL was most effective.

### Effects of *R. mucilaginosa* in Combination with PA on the Mycelial Growth of *P. expansum*

**FIGURE 3:**
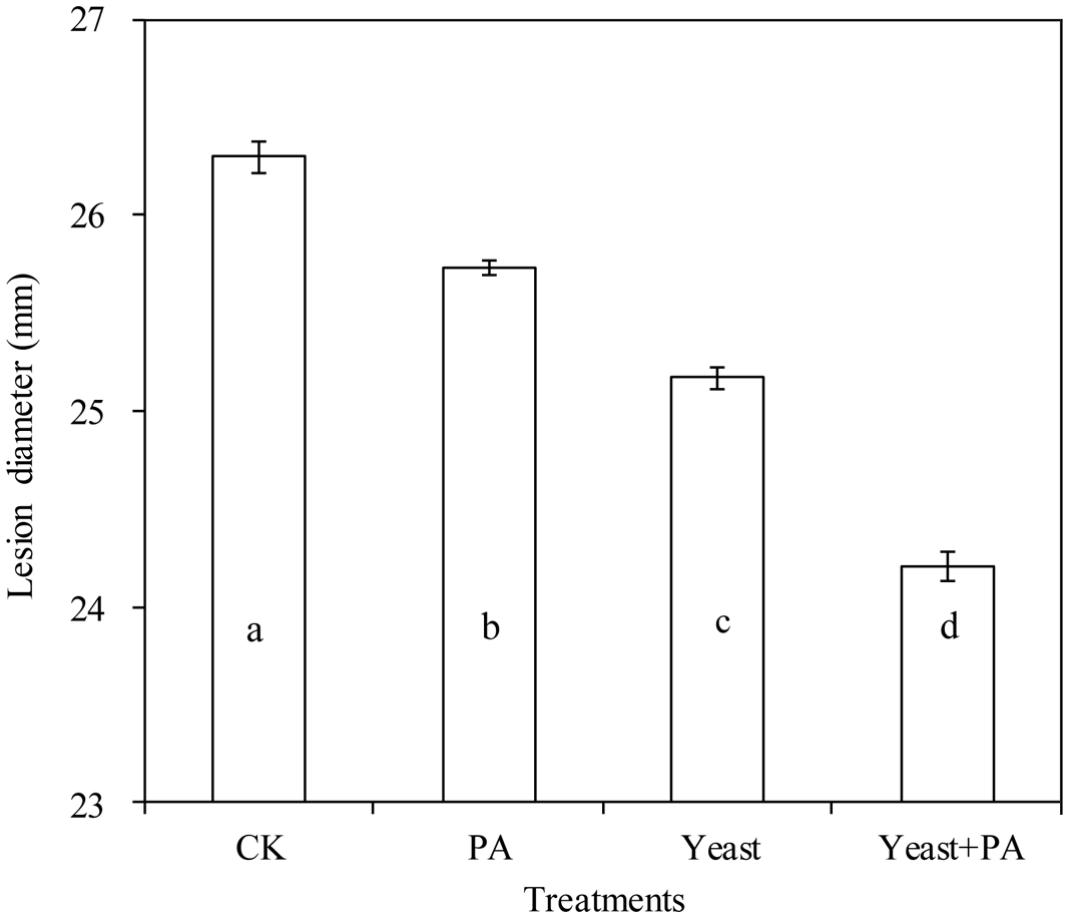
**Effects of *R. mucilaginosa* in combination with PA on mycelial growth *of Penicillium expansum*.** Treatments are as follows: CK = sterile distilled water, PA = 4 μmol/mL PA, yeast = *R. mucilaginosa* (1 × 10^8^ CFU/mL), yeast + PA = *R. mucilaginosa* (1 × 10^8^ CFU/mL) + 4 μmol/mL PA. Each value is the mean of three experiments. Bars represent standard deviations. Different letters indicate significant differences (*P* < 0.05) according to the Duncan’s multiple range test.

On PDA plates, 4 μmol/mL PA, and *R. mucilaginosa*as as stand-alone treatments with diameters of 25.73 and 25.17 cm, inhibited the growth of *P. expansum*, compared to the control (26.30 cm; **Figure 3**). However, the efficacy of the combined treatment of *R. mucilaginosa* and 4 μmol/mL PA with diameter of 24.21 cm, was better than *R. mucilaginosa* or PA as stand-alone treatments.

### Effects of *R. mucilaginosa* in Combination with PA on Patulin Production by *P. expansum* in NYDB Media

**FIGURE 4:**
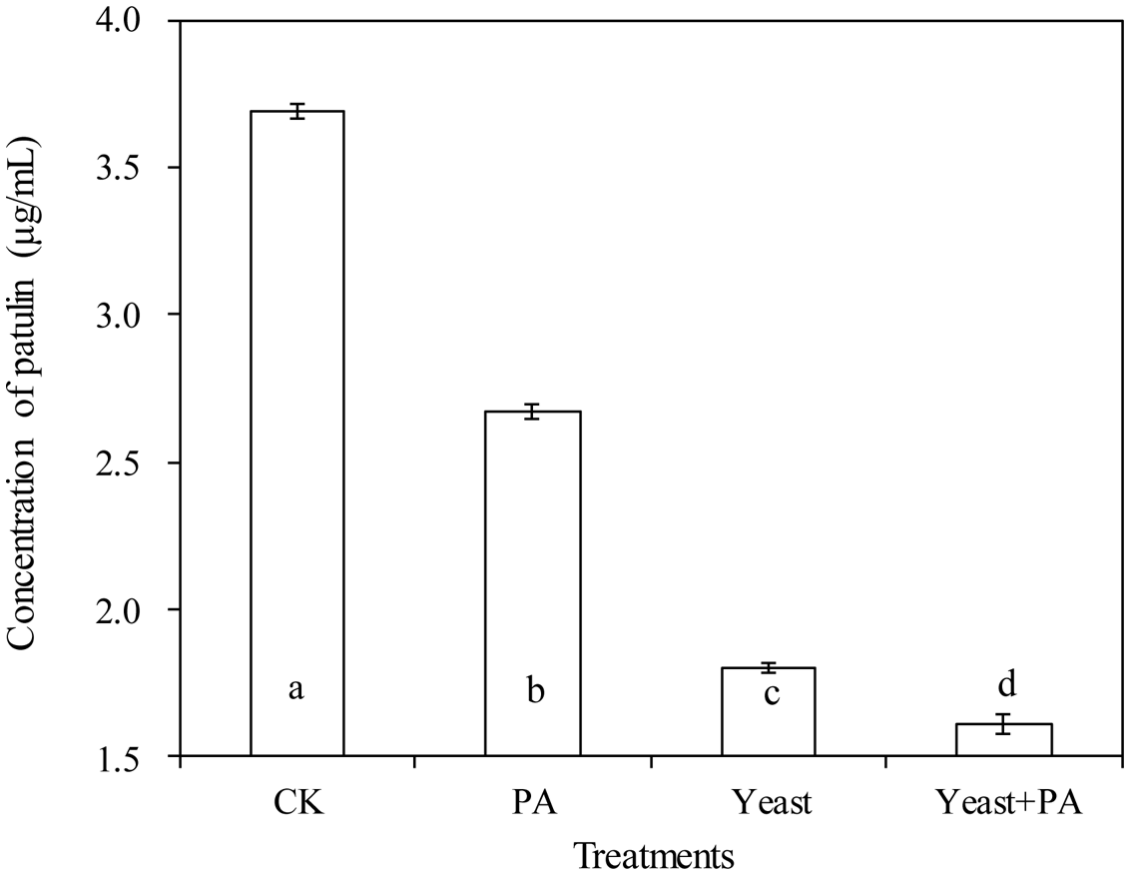
**Effects of *R. mucilaginosa* in combination with PA on patulin content produced by *P. expansum* in NYDB media.** Treatments are as follows: CK = sterile distilled water, PA = 4 μmol/mL PA, yeast = *R. mucilaginosa* (1 × 10^8^ CFU/mL), yeast + PA = *R. mucilaginosa* (1 × 10^8^ CFU/mL) + 4 μmol/mL PA. Each value is the mean of two experiments. Bars represent standard deviations. Different letters indicate significant differences (*P* < 0.05) according to the Duncan’s multiple range test.

**FIGURE 5:**
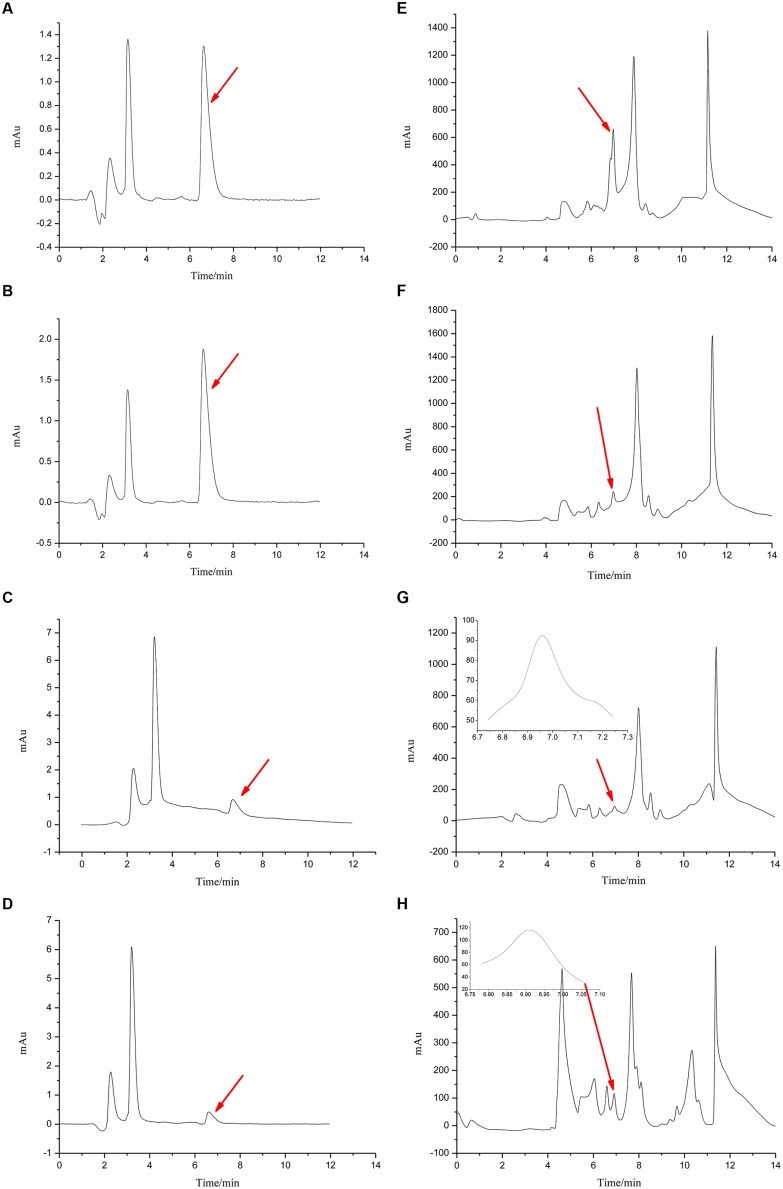
**Chromatograms for **Figures 4** and **6**.** The chromatograms from **(A–D)** show the effects of *R. mucilaginosa* in combination with PA on patulin content produced by *P. expansum* in NYDB media. The chromatograms from **(E–H)** show the effects *R. mucilaginosa* in combination with PA on patulin content produced by *P. expansum* in apples. Treatments are as follows: **(A)**, E = sterile distilled water, **(B,F)** = 4 μmol/mL PA, **(C,G)** = *R. mucilaginosa* (1 × 10^8^ CFU/mL), **(D,H)** = *R. mucilaginosa* (1 × 10^8^ CFU/mL) + 4 μmol/mL PA.

Phytic acid (4 μmol/mL) and *R. mucilaginosa* as stand-alone treatments significantly inhibited patulin production by *P. expansum* in NYDB media (**Figures 4** and **5A–D**). The concentration of patulin was 2.671 and 1.798 μg/mL. However, *R. mucilaginosa* in combination with PA had greater inhibition on patulin production by *P. expansum* (1.607 μg/mL) than the *R. mucilaginosa* or PA treatments alone.

### Effects of *R. mucilaginosa* in Combination with PA on the Content of Patulin Produced by *P. expansum* in Apples

**FIGURE 6:**
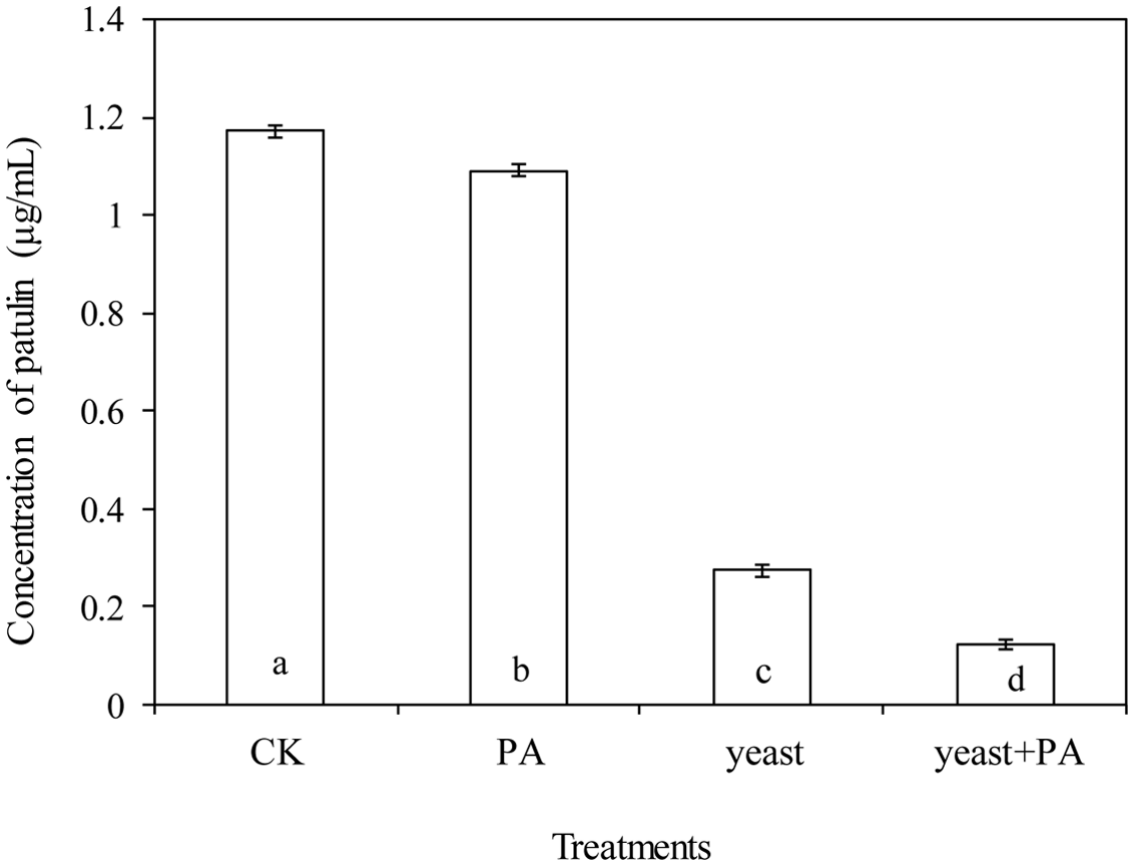
**Effects of *R. mucilaginosa* in combination with PA on the patulin content produced by *P. expansum* in apples.** Fruit treatments are as follows: CK = sterile distilled water, PA = 4 μmol/mL PA, yeast = *R. mucilaginosa* (1 × 10^8^ CFU/mL), yeast + PA = *R. mucilaginosa* (1 × 10^8^ CFU/mL) + 4 μmol/mL PA. Bars represent standard deviations. Different letters indicate significant differences (*P* < 0.05) according to the Duncan’s multiple range test.

Phytic acid (4 μmol/mL) and *R. mucilaginosa*, used alone significantly decreased the content of patulin in apple wounds produced by *P. expansum* in by 6.9 and 76.8% respectively, compared to the control (**Figures 5E–H** and **6**). However, *R. mucilaginosa* in combination with PA, had even greater inhibition than PA and *R. mucilaginosa* used alone, decreasing the content of patulin by 89.6% compared to the control.

### Degradation of Patulin by *R. mucilaginosa*, used Alone or in Combination with PA *In Vitro*

The initial patulin concentration of 20 μg/mL in the presence of *R. mucilaginosa* cells was decreased over 92.11% after 24 h incubation at 28°C; the final patulin concentration was 1.578 μg/mL (**Table 1**). Similarly, *R. mucilaginosa* in combination with PA (4 μmol/mL) decreased the initial patulin concentration of 20–1.561 μg/mL, and the degradation rate was 92.2%.

**Table 1:** The degradation of patulin by *R. mucilaginosa*, used alone or in combination with PA *in vitro*.

Treatments	Initial patulin concentration (μg/mL)	Final patulin concentration (μg/mL)	Degradation rate (%)
*R mucilaginosa*	20	1.578	92.1
*R. mucilaginosa +* 4 μmol/mL P	20	1.561	92.2


### Effects of PA at Various Concentrations on Antagonistic Yeast

**FIGURE 7:**
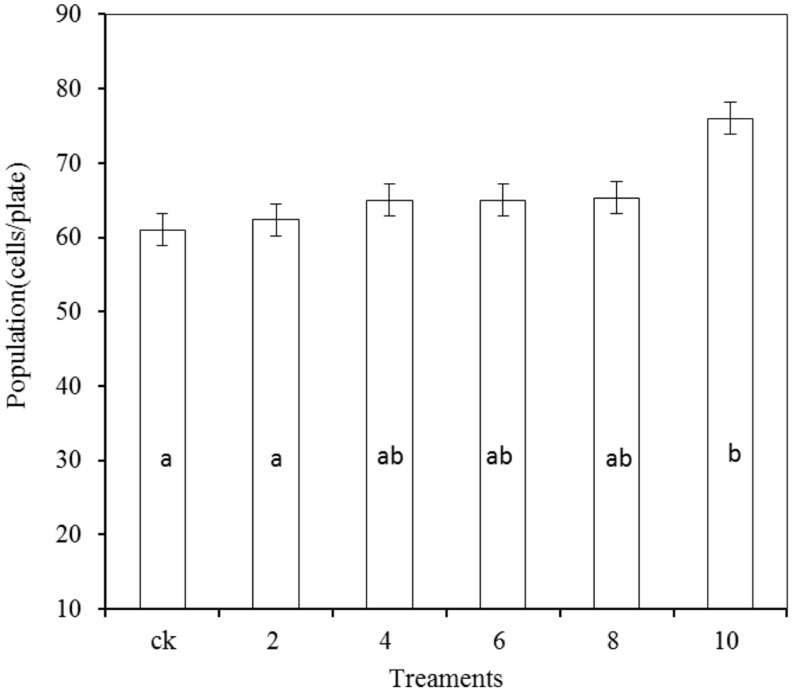
**Effects of PA at various concentrations on antagonistic yeast.** Treatments are as follows: CK = *R. mucilaginosa* (1 × 10^4^ CFU/mL), (2, 4, 6, 8, 10) = *R. mucilaginosa* (1 × 10^4^ CFU ml^-1^) + PA at different concentrations (2, 4, 6, 8, and 10 μmol/mL). Each value is the mean of two experiments. Bars represent standard deviations. Different letters indicate significant differences (*P* < 0.05) according to the Duncan’s multiple range test.

As shown in **Figure 7**, the growth of *R. mucilaginosa* in combination with PA at concentrations of 2, 4, 6, and 8 μmol/mL showed no significant effect compared with the stand-alone *R. mucilaginosa* treatment. The combined treatment of *R. mucilaginosa* and PA at the concentration of 10 μmol/mL was most effective.

## Discussion

Natural antioxidants extracted from various plants and fungi have been used recently as novel compounds in the battle against post-harvest development of fungi and the production of mycotoxins (i.e., aflatoxins, ochratoxin A; Ricelli et al., 2002; Reverberi et al., 2005; Zjalic et al., 2006). Recently, phytic acid has been considered to be an antioxidant agent, as it is a potent inhibitor of iron-catalyzed hydroxy radical formation, by chelating the free iron and then blocking the iron coordination site (Graf and Eaton, 1990). In our study, PA at various concentrations, significantly decreased the disease incidence of blue mold decay in apples caused by *P. expansum*. The results reported here indicate that PA at an appropriate concentration could control postharvest blue mold decay of apples.

The use of biocontrol agents to manage the postharvest decay of fruit has been explored as an alternative to the use of synthetic fungicides (Wilson and Wisniewski, 1989). Our results showed that *R. mucilaginosa* as stand-alone treatment significantly reduced the disease incidence of blue mold decay of apples stored at 20°C. This suggests that *R. mucilaginosa* has the potential as a biocontrol agent in the control of postharvest blue mold decay of apples. The combination treatment of *R. mucilaginosa* with PA at 4 μmol/mL also significantly reduced the disease incidence of blue mold decay and the lesion diameter of apples at 20°C, with greater efficacy than *R. mucilaginosa* alone. This indicates that PA at appropriate concentration can enhance the biocontrol efficacy of *R. mucilaginosa*. The mechanism by which PA enhances the biocontrol efficacy of yeast is complex, but it may be attributed to the influence of PA on the antagonist, pathogen, and host. We found that PA used alone, at 2 and 10 μmol/mL, had little effect on apple decay. Perhaps PA used as antioxidant, does not enhance the antagonistic yeast at low or high concentrations.

Under *in vitro* conditions, PA and *R. mucilaginosa* as stand-alone treatments, inhibited the growth of *P. expansum* on PDA plates, and the efficacy of the combined treatments of *R. mucilaginosa* and PA was better than that of the *R. mucilaginosa* standalone treatment. These results show that PA has the ability to enhance the inhibitory efficacy of *R. mucilaginosa* on the growth of *P. expansum*, and this may be one of the mechanisms by which PA has the ability to enhance the biocontrol efficacy of yeast on postharvest blue mold decay of apples.

Many studies have shown that some yeast antagonists can directly inhibit the production of patulin, while inhibiting *P. expansum* growth. Coelho et al. (2007) found that an initial patulin content of 223 μg in the presence of 158 *P. ohmeri* cells decreased 83% when incubated at 25°C for 2 days, and decreased >99% after 5 days incubation. Patulin levels were undetectable after 15 days. The pH decreased from 4.0 to 3.3 during the 15 days experiment, suggesting that the patulin decrease was an active process and a consequence of yeast metabolism. Our results showed that *R. mucilaginosa* as stand-alone treatments significantly inhibited patulin production by *P. expansum* in NYDB media. Additionally, *R. mucilaginosa* alone significantly decreased the content of patulin in apple wounds produced by *P. expansum*. These results indicate that *R. mucilaginosa* can control the accumulation of patulin produced by *P. expansum* in apples. Similarly with the biocontrol efficacy of antagonistic yeasts on postharvest diseases, the efficacy of antagonistic yeasts in controlling the accumulation of patulin in apples needs to be enhanced. Tolaini et al. (2010), found that the biocontrol effects of *Cryptococcus laurentii*, used together with *Lentinula edodes*, in wounded apples improved the inhibition of *P. expansum* growth and patulin production compared to *C. laurentii* alone, under both experimental and semi-commercial conditions. The relationship between oxidative stress and mycotoxin biosynthesis was further confirmed using antioxidants from *Fusarium graminearum* and *F. culmorum* (Reverberi et al., 2010). The metabolic requirement of resistance to a heavily oxidized environment can be a limiting factor for a potential biocontrol agent. Tolaini et al. (2010) described the role of *L. edodes* culture filtrates in reinforcing the competitiveness of the biocontrol agent *C. laurentii* through the enhancement of its antioxidative potential. Similarly, the use of plant-derived antioxidants such as caffeic acid, flavonoids, and phenolic acid from tree nuts reduces Aflatoxigenesis (AF) production by *A. flavus* by up to 99%, without affecting fungal growth (Mahoney et al., 2010). The antioxidant function of PA is different from that of other antioxidants, as part of the antioxidative effects of PA in lipid-containing model systems can be explained by its radical-scavenging activity. The results from this study show that yeast culture supernatant with 4 μmol/mL PA exhibited greater inhibition of patulin production by *P. expansum* than either *R. mucilaginosa* or PA treatments alone, suggesting that PA could enhance the inhibitory activity of *R. mucilaginosa* on the production of patulin. This may be because PA can enhance the inhibitory efficacy of *R. mucilaginosa* on the growth of *P. expansum.* Furthermore, the combination of *R. mucilaginosa* and PA could significantly reduce the patulin content of apples, produced by *P. expansum*. The results obtained suggest that the use of PA (4 μmol/mL) together with *R. mucilaginosa* improves the efficiency of the biocontrol activity of the yeast, leading to an almost total inhibition of *P. expansum* growth and patulin production in apples.

In the degradation of patulin by *R. mucilaginosa*, alone or in combination with PA *in vitro*, the initial patulin concentration of 20 μg/mL in the presence of *R. mucilaginosa* cells was decreased 92.1%, when incubated at 28°C for 24 h. Similarly, the initial patulin concentration of 20 μg/mL in the presence of *R. mucilaginosa* with 4 μmol/mL PA decreased over 92.2%. This shows that PA could not enhance the patulin degradation activity of *R. mucilaginosa*, however, the degradation efficacy of *R. mucilaginosa* or *R. mucilaginosa* in combination with PA is high, and the patulin was almost completely degraded after 24 h.

## Conclusion

Phytic acid at a concentration of 4 μmol/mL significantly enhanced the biocontrol activity of *R. mucilaginosa* against postharvest blue mold decay and the production of patulin in apples. The mode of action may be its direct inhibition on the colony growth of the pathogens. Additionally, both *R. mucilaginosa* and *R. mucilaginosa* in combination with PA degrade patulin *in vitro*. The effective antagonism of *R. mucilaginosa* versus *P. expansum* hyphal growth suggested that the inhibition of fungal development is a promising alternative for biological control, and consequent potential for low patulin levels in apples. Future research will be aimed at developing the technology to be used in large-scale operations and further investigating the mode of action of PA’s enhancement of the biocontrol efficacy of *R. mucilaginosa* on postharvest blue mold decay and patulin production in apples.

## Author Contributions

HZ, QY, and XZ conceived and designed the experiments. QY, XZ, HZ, and XZ performed the experiments. JQ analyzed the data. HZ, QY, and JQ drafted the manuscript. All authors read and approved the final manuscript.

## Conflict of Interest Statement

The authors declare that the research was conducted in the absence of any commercial or financial relationships that could be construed as a potential conflict of interest.
